# Quantitative Assessment of Liver Function Using the Liver Enhancement Ratio on Gadoxetic Acid–Enhanced Magnetic Resonance Imaging in Chronic Liver Disease

**DOI:** 10.5152/tjg.2026.25827

**Published:** 2026-06-03

**Authors:** Betul Akdal Dolek, Halil Tekdemir, Sercan Sahin, Eren Camur, Ali Haydar Baykan, Rıza Sarper Okten

**Affiliations:** 1 Department of Radiology, Ankara Bilkent City Hospital, Ankara, Türkiye; 2 Department of Radiology, Ankara Etlik City Hospital, Ankara, Türkiye; 3 Department of Radiology, Ayvalık Hospital, Balıkesir, Türkiye; 4 Department of Radiology, 29 Mayıs Hospital, Ankara, Türkiye; 5 Department of Radiology, Adıyaman University, Adıyaman, Türkiye

**Keywords:** Chronic liver disease, fibrosis, hepatobiliary contrast agents, liver function tests, magnetic resonance imaging

## Abstract

**Background/Aims:**

Noninvasive assessment of hepatic functional reserve remains a clinical challenge in patients with chronic liver disease (CLD). This study aimed to quantify the association between liver enhancement ratio (LER) and standard biochemical liver function scores and to determine clinically meaningful cutoff values for functional stratification.

**Materials and Methods:**

This retrospective study included 187 subjects (78 with CLD and 109 controls) who underwent gadoxetic acid–enhanced magnetic resonance imaging (MRI). Signal intensities from 5 liver segments were measured on precontrast and hepatobiliary-phase images to calculate LER. LER values were compared between groups and across liver function categories using nonparametric tests and correlated with model for end-stage liver disease (MELD), albumin–bilirubin (ALBI), and fibrosis-4 (FIB-4) scores.

**Results:**

LER was significantly lower in the CLD group than in controls (*P* < .001). The optimal threshold for discriminating CLD from normal hepatic parenchyma was 1.01, with an area under the curve (AUC) of 0.83 (95% CI, 0.76-0.89). As hepatic functional impairment progressed, LER demonstrated a stepwise decline and showed moderate inverse correlations with MELD (*ρ* = −0.439, *P* < .001), ALBI (*ρ* = −0.579, *P* < .001), and FIB-4 (*ρ* = −0.421, *P* < .01). For identification of ALBI grade 2 or higher, an LER cutoff of 0.72 yielded an AUC of 0.84 (95% CI, 0.77-0.89). For prediction of MELD scores of 10 or higher, an LER threshold of 0.62 yielded an AUC of 0.73.

**Conclusion:**

LER is a reproducible quantitative MRI biomarker that demonstrates moderate correlations with established biochemical and clinical measures of liver function and provides clinically interpretable cutoff values for CLD detection and functional grading.

**Main Points**

- Liver enhancement ratio (LER) is significantly lower in patients with chronic liver disease (CLD) than in healthy controls.
- LER demonstrates moderate inverse correlations with model for end-stage liver disease (MELD), albumin–bilirubin (ALBI), and fibrosis-4.
- LER demonstrates a progressive decline across worsening Child–Pugh, ALBI, and MELD categories.
- An LER cutoff of 1.01 discriminates patients with CLD from controls, with an AUC of 0.83.

## Introduction

Chronic liver disease (CLD) represents a substantial global health burden and arises from diverse etiologies such as chronic viral hepatitis, metabolic dysfunction–associated steatotic liver disease, alcohol-associated liver injury, and immune-mediated hepatopathies.^1^^-^^3^ In combination with other liver diseases, nearly 2 million people are estimated to die annually from liver failure.^4^^,^^5^ CLD is characterized by gradual parenchymal remodeling with progressive fibrosis and a stepwise decline in hepatic synthetic and metabolic function. In clinical practice, disease severity and prognostic stratification are commonly assessed using validated laboratory-based scoring system, including Child–Pugh, albumin–bilirubin (ALBI), and the model for end-stage liver disease (MELD).^6^^-^^8^ However, reliable, reproducible, and noninvasive imaging biomarkers are still needed because traditional indices are subject to laboratory variability and may not accurately reflect hepatic spatial or functional heterogeneity.^9^^,^^10^

Among hepatic imaging modalities, magnetic resonance imaging (MRI) provides superior soft-tissue contrast and quantitative capability compared with ultrasound or computed tomography (CT).^11^^-^^13^ There is particular interest in MRI using the hepatocyte-specific contrast agent gadoxetic acid because of its ability to provide both morphologic and functional information. Gadoxetic acid is taken up by functioning hepatocytes via OATP1B1/1B3 transporters and excreted into bile via MRP2 channels, resulting in increased liver signal intensity (SI) during the hepatobiliary phase.^14^^-^^16^ Because this enhancement reflects hepatocellular uptake and excretory capacity, gadoxetic acid–enhanced MRI offers a noninvasive approach for assessing hepatic functional reserve. The liver enhancement ratio (LER), defined as the relative increase in the liver SI between precontrast and hepatobiliary-phase (HBP) images, has been proposed as an indicator of liver function. Early studies have demonstrated that LER differentiates normal from diseased liver parenchyma and correlates with biochemical liver function scores.^17^^-^^19^ However, diagnostic thresholds and clinical utility of LER remain unresolved in quantitative liver imaging.

This study aimed to address this gap by quantifying the relationship between LER and standard liver function scores and determining clinically useful LER cutoff values for disease detection and functional grading.

## Materials and Methods

### Study Design

**Figure 1.**
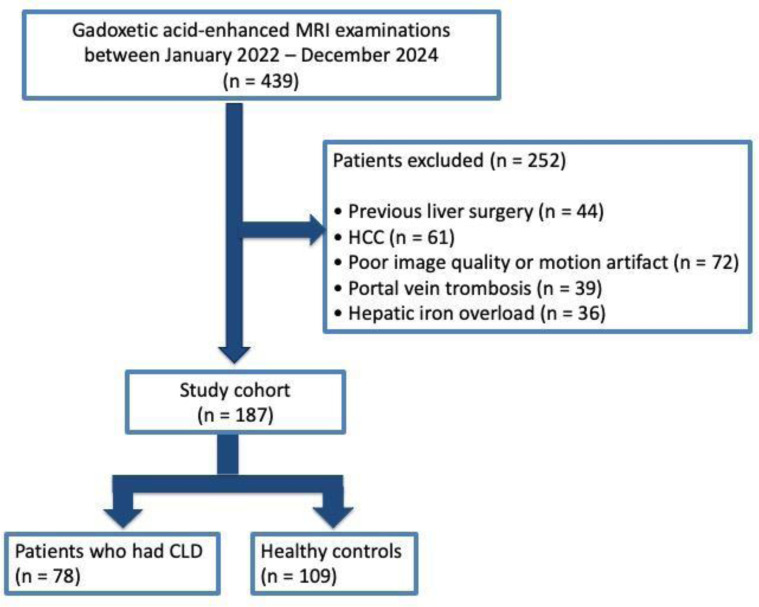
Flowchart of patient selection.

This study was conducted after approval from the institutional ethics committee of Ankara Bilkent City Hospital (approval number: TABED 2-24-70, approval date: March 20, 2024). Written informed consent was waived due to the retrospective nature of the study and in accordance with institutional ethics committee approval. No artificial intelligence tools were used in the preparation of this article. A total of 439 consecutive patients who underwent gadoxetic acid–enhanced MRI between January 2022 and January 2024 were initially reviewed. Inclusion criteria were availability of laboratory data obtained within 1 week of the MRI examination and sufficient image quality for quantitative analysis. Of the initial 439 patients, 252 (57.4%) were excluded for the following reasons: prior liver surgery (n = 44), hepatocellular carcinoma (n = 61), portal vein thrombosis (n = 39), hepatic iron overload (n = 36), and insufficient image quality (n = 72). Hepatic iron overload was defined as low liver SI on in-phase and out-of-phase T1-weighted and T2-weighted images consistent with iron deposition in the radiology report and/or a prior clinical diagnosis of hemochromatosis or secondary iron overload. After application of these criteria, 187 patients were included in the final analysis. The study workflow is presented in Figure 1.

In the control group, nonhepatic MRI indications included adrenal evaluation, pancreatic or biliary lesions, renal or retroperitoneal abnormalities, and follow-up of indeterminate nonhepatic findings. Control subjects were required to have no known CLD, normal liver biochemistry (bilirubin, alanine aminotransferase (ALT), aspartate aminotransferase (AST), alkaline phosphatase, and γ-glutamyltransferase), and no imaging evidence of hepatic steatosis, fibrosis, or portal hypertension on ultrasound, CT, or MRI. Electronic medical records and medication lists were systematically reviewed to exclude control subjects with significant alcohol intake, chronic hepatotoxic medication use, or abnormal liver biochemistry and to classify patients with CLD according to underlying etiology.

### Clinical and Laboratory Data

Baseline characteristics included age, sex, body mass index (when documented), alcohol consumption, underlying CLD etiology, and relevant comorbid conditions (e.g., diabetes mellitus and hypertension). Biochemical and hematologic data—including total bilirubin, serum albumin, international normalized ratio (INR), creatinine, AST, ALT, and platelet count—were collected to calculate Child–Pugh, MELD, ALBI, and fibrosis-4 (FIB-4) scores.

The Child–Pugh classification was based on serum albumin, total bilirubin, INR, and the presence and severity of ascites and hepatic encephalopathy. Participants were stratified into class A (5-6 points), class B (7-9 points), or class C (≥10 points). The ALBI score was calculated using the equation (0.66 × log10 bilirubin) + (−0.085 × albumin), and ALBI grade was assigned as follows: grade 1 for scores ≤ −2.60, grade 2 for scores between −2.60 and −1.39, and grade 3 for scores > −1.39.^20^ The FIB-4 index was calculated as (age × AST)/(platelet count × √ALT). Consistent with prior literature, a cutoff of 1.45 was applied, as values below this threshold have been reported to have an approximately 90% negative predictive value for ruling out advanced fibrosis.^21^^,^^22^ The MELD score was derived using 9.57 × log(creatinine [mg/dL]) + 3.78 × log(bilirubin [mg/dL]) + 11.2 × log(INR) + 6.43.^23^

All laboratory measurements (albumin, bilirubin, creatinine, INR, ALT, AST, and platelet count) were extracted from the electronic medical record within 7 days of the MRI examination. These clinical and laboratory variables were subsequently used for patient stratification and comparative and correlation analyses.

### Magnetic Resonance Imaging Acquisition and Image Analysis

All MRI scans were obtained on a 3.0-T system (SIGNA, GE HealthCare, Waukesha, WI, USA) using a phased-array abdominal coil with patients in the supine position. Axial fat-suppressed T1-weighted images were acquired before contrast administration. HBP images were then acquired 20 minutes after intravenous injection of gadoxetic acid (Primovist, Bayer Healthcare, Berlin, Germany) at a dose of 0.025 mmol/kg followed by a 20-mL saline flush.

HBP images were acquired using a 3D LAVA-Flex (liver acquisition with volume acceleration–flex) spoiled gradient-echo sequence with the following parameters: repetition time, 3.4 ms; echo time, 1.7 ms; flip angle, 15°; matrix, 256 × 192; field of view, 36-40 cm; and slice thickness, 3 mm. All acquisitions were performed during the end-expiration breath-hold.Automatic B_0_ and B_1_ shimming was applied before scanning to minimize field inhomogeneity and improve signal uniformity.

**Figure 2.**
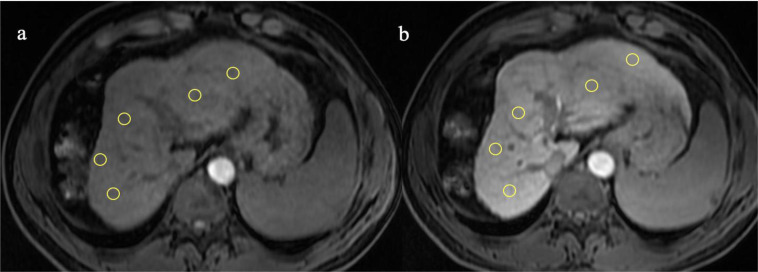
Regions of interest (ROI) placement for liver enhancement ratio measurement on gadoxetic acid-enhanced magnetic resonance imaging. Circular ROIs were placed in identical locations on both precontrast and hepatobiliary-phase images, covering both hepatic lobes and avoiding vascular and biliary structures.

SI values were measured on precontrast and HBP T1-weighted images. Five circular regions of interest (ROIs), each measuring approximately 1-2 cm^2^, were placed in different hepatic segments (3 in the right lobe and 2 in the left lobe) while avoiding larger vessels and bile ducts (Figure 2). ROIs were placed away from the liver margins to avoid partial-volume effects and were replicated at identical anatomic locations on both precontrast and HBP images. The mean SI value of these ROIs in both phases was calculated. The LER was calculated via the following formula:

LER = (SI_HBP20 − SI_pre)/SI_pre

Two abdominal radiologists with 8 and 11 years of experience in abdominal MRI independently placed ROIs and calculated LER values, blinded to all clinical and laboratory data. For statistical analysis, the mean of the 2 readers’ LER measurements was used.

### Statistical Analysis

Analyses were performed using SPSS version 24.0 (IBM SPSS Corp.; Armonk, NY, USA). The Shapiro–Wilk test was used to assess data distribution. Given non-Gaussian data, comparisons between 2 groups were performed using the Mann–Whitney *U*-test, whereas comparisons among 3 or more groups were performed using the Kruskal–Wallis test. When appropriate, Bonferroni-adjusted post hoc pairwise comparisons were conducted. Associations between LER and clinical severity metrics (Child–Pugh, MELD, ALBI, and FIB-4) were assessed using Spearman’s rank correlation (*ρ*). Receiver operating characteristic (ROC) curve analyses were performed to derive LER thresholds for discriminating CLD from controls and for categorizing functional severity according to Child–Pugh and ALBI; optimal cut points were selected using the Youden index. Interreader agreement was assessed using a 2-way random-effects intraclass correlation coefficient (ICC) with 95% CIs. All hypothesis tests were 2-sided, with *P* < .05 considered statistically significant.

## Results

A total of 187 subjects were enrolled: 78 patients with CLD (41.7%) and 109 healthy controls (58.3%). The median age was 57.5 years (IQR, 47.0-62.8) in the CLD patients and 51.0 years (IQR, 42.0-59.0) in the control group (*P* = .17). Female patients accounted for 42.3% (n = 33) of the CLD group and 55.0% (n = 60) of the control group (Table 1).

The most common etiology in the CLD group was hepatitis B virus infection (29 patients, 37.2%), followed by nonalcoholic fatty liver disease (NAFLD) (18 patients, 23.1%) and hepatitis C virus infection (11 patients, 14.1%).

**Figure 3.**
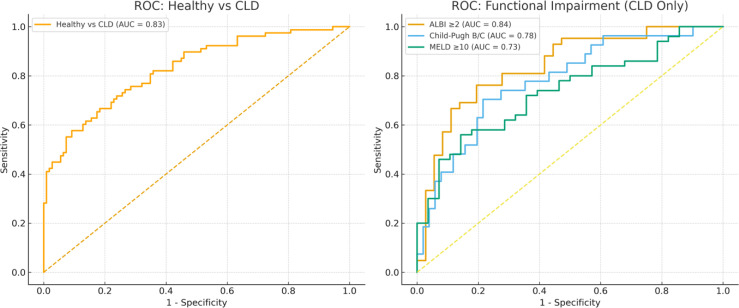
Receiver operating characteristic (ROC) curves of the liver enhancement ratio (LER). (A) ROC analysis for differentiating healthy subjects from patients with chronic liver disease (AUC = 0.83). (B) ROC analyses for functional impairment defined as ALBI grade ≥2 (AUC = 0.84), Child–Pugh class B/C (AUC = 0.78), and MELD score ≥10 (AUC = 0.73).

**Figure 4.**
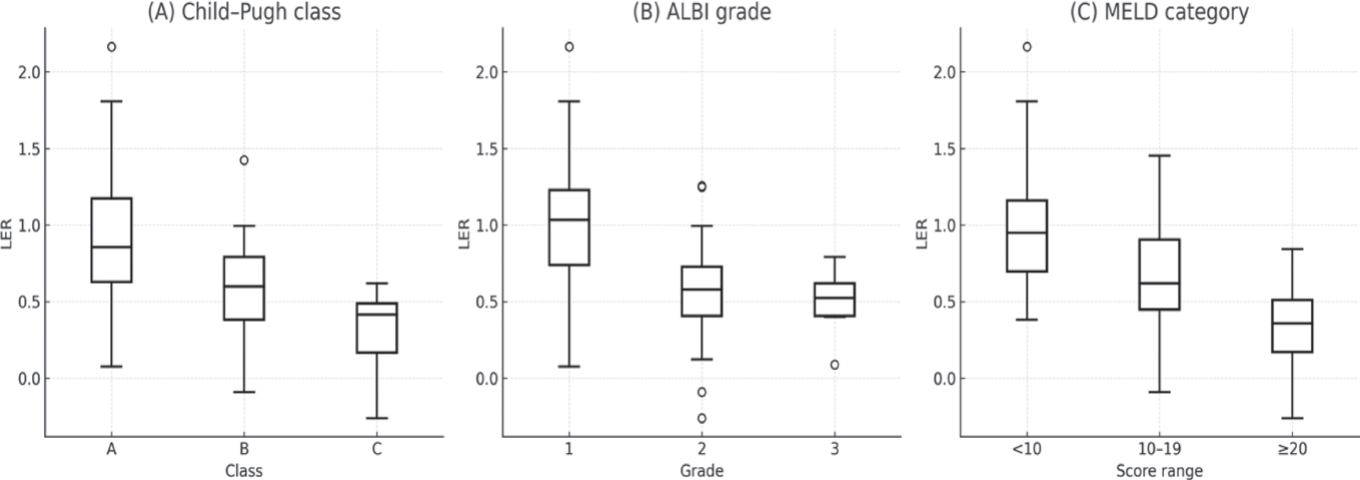
Boxplots of liver enhancement ratio (LER) across liver function classifications. (A) Child–Pugh classes (A, B, and C), (B) ALBI grades (1, 2, and 3), and (C) MELD categories (<10, 10-19, and ≥20).

The laboratory and clinical data of patients with CLD are presented in Table 2. The median MELD score was 11 (range, 9-23). According to the Child–Pugh classification, 51 patients were categorized as class A, 21 as class B, and 6 as class C. Patients with CLD had significantly lower LER values than healthy controls (*P* < .001). Median LER values in controls and CLD subgroups according to Child–Pugh class, ALBI grade, and MELD category are presented in Table 3. Interobserver agreement for LER measurements was excellent (ICC = 0.91). For distinguishing CLD from healthy controls, the optimal LER cutoff was 1.01, with an AUC of 0.83 (95% CI, 0.76-0.89), sensitivity of 57.7% (95% CI, 46.2-68.7%) and specificity of 90.8% (95% CI, 83.6-95.2%) (Table 4, Figure 3A). LER values decreased progressively with worsening hepatic function according to the Child–Pugh classification (Figure 4).

Notably, significant differences were observed between Child–Pugh classes A and B (*P* < .01) and between classes A and C (*P* = .012), whereas no difference was observed between classes B and C. Using ROC analysis, an LER cutoff of 0.62 discriminated Child–Pugh B/C from A with an AUC of 0.78 (95% CI, 0.69-0.85), sensitivity of 70.4% (95% CI, 55.3-82.9), and specificity of 78.4% (95% CI, 65.3-88.1) (Table 4, Figure 3B). A lower cutoff of 0.522 specifically identified Child–Pugh class C compared with classes A/B, with an AUC of 0.86. When Child–Pugh classes were grouped as compensated (A) vs. decompensated (B/C), LER distinguished these groups with an AUC of 0.78 using a cutoff of 0.62.

There was a moderate inverse correlation between LER and MELD score (*ρ* = −0.439, *P* < .001). For descriptive purposes, patients were stratified into 3 MELD categories: <10, 10-19, and ≥20. LER values declined progressively across these groups, with median LER values of 0.95 in patients with MELD <10, 0.62 in those with MELD 10-19, and 0.36 in patients with MELD ≥20. For ROC analysis, MELD was dichotomized at ≥10, and after correcting the direction of the enhancement metric, the inverted LER yielded an AUC of 0.73 with an optimal cutoff equivalent to an LER value of 0.62 (sensitivity 56% and specificity 86%). LER values were significantly lower in patients with MELD ≥20 than in those with MELD <10 (*P* = .009). A moderate inverse correlation was also observed between the LER and the FIB-4 index (*ρ* = −0.421, *P* < .01) in patients with CLD. Although exploratory, the ROC analysis for FIB-4 index ≥1.45 provided an LER cutoff of 0.968 (AUC = 0.71), indicating moderate diagnostic accuracy.

When stratified by ALBI grade, significant differences in LER were observed between grades 1 and 2 (*P* < .01) and between grades 1 and 3 (*P* < .01), indicating that the LER decreases with worsening hepatic function (Figure 4). No significant difference was observed between ALBI grades 2 and 3.

Correlation coefficients between LER and liver function scores are presented in Table 5.

## Discussion

The study revealed that LER demonstrated moderate correlations with biochemical parameters and a stepwise decline corresponding to worsening liver function scores. An LER cutoff level of 1.01 (AUC = 0.83) differentiated patients with CLD from healthy controls.

Despite these statistically significant associations, LER demonstrated only moderate diagnostic performance, with sensitivities of 57.7% for CLD and 56.0% for MELD ≥10, and no significant differences were observed for Child–Pugh classes B and C. These findings limit the performance of LER as a standalone decision-making tool in patients with advanced hepatic dysfunction.

The study provides additional evidence by defining specific LER cutoff values for CLD detection and functional grading in a single-center cohort. Collectively, these findings suggest that LER may be most useful as an adjunctive tool in intermediate-risk patients rather than as a primary screening or definitive diagnostic test. In cases where CLD is suspected but conventional scores are indeterminate, LER could help identify patients who may benefit from closer follow-up or referral to a specialist.

Laboratory-derived indices such as FIB-4 are susceptible to modulation by systemic inflammation, nutritional status, and extrahepatic comorbidities, which may limit their specificity for hepatic functional reserve and fibrosis burden. In contrast, LER obtained from gadoxetic acid–enhanced MRI reflects hepatocellular uptake and biliary excretion, providing a more liver-specific and physiology-based assessment.^24^^,^^25^ In the cohort, LER decreased stepwise with worsening ALBI grade and Child–Pugh class and provided clinically interpretable thresholds for functional stratification, supporting its potential value for objective severity grading and individualized follow-up planning. Although the inverse association between LER and FIB-4 was only moderate, it suggests that impaired hepatocellular contrast handling may also parallel structural injury, supporting the concept that LER can complement conventional laboratory scores by integrating functional and, to some extent, fibrosis-related information into routine practice.^24^^-^^26^

Previous studies have similarly used HBP signal–based parameters to distinguish healthy from diseased liver parenchyma. Lee et al^27^ reported that an signal intensity change (SIC) value of 2.17 (AUC = 0.76) distinguished CLD from normal liver using a liver-only correction method. Verloh et al^24^ reported a relative improvement threshold of 47.7% (AUC = 0.87) for advanced fibrosis, whereas Beer et al^17^ and Takatsu et al^28^ reported strong correlations between the HBP index and the Child–Pugh class (AUC = 0.85-0.89). Eryuruk et al^29^ subsequently validated an MRI-derived relative enhancement index, which is conceptually related to LER, showed a strong correlation with ALBI grade, and demonstrated superiority over the functional liver imaging score. However, metrics using liver-to-spleen or liver-to-muscle ratios are susceptible to confounders such as splenomegaly and systemic illness.^30^ In contrast, the study used a self-referenced hepatic-only LER, thereby minimizing scanner- and extrahepatic related variability, which is consistent with recent harmonization trends.^31^

In addition to disease detection, the validated LER thresholds enabled effective functional grading. The inverse correlation between LER and MELD score (*ρ* = −0.439, *P* < .001) further supports its use as a noninvasive surrogate marker of hepatic reserve. LER demonstrated a moderate inverse correlation with ALBI grade (*ρ* = −0.58, *P* < .001) and yielded an AUC of 0.84, with an optimal cutoff of 0.72 for identifying ALBI grade ≥ 2. These findings indicate that LER functions as a continuous, quantitative index of hepatocellular function that can be applied across multiple scoring systems.^17^^,^^24^^,^^28^

The reduction in LER observed in CLD reflects impaired hepatocellular uptake mediated by OATP1B1/1B3 transporters and reduced excretion via MRP2 during fibrogenesis.^32^^-^^36^ These molecular processes support the biological link between hepatocyte function and gadoxetic acid enhancement. The quantitative assessment of LER thereby provides a physiologically based measure of hepatocellular transport capacity. Compared with systemic biomarkers, LER is less affected by extrahepatic factors such as systemic inflammation and portal congestion.

Since many patients with CLD already undergo gadoxetic acid–enhanced MRI for lesion detection or structural assessment, LER can be incorporated into routine protocols to provide incremental functional information without additional scan time or contrast administration. Clinically, LER appears most useful in patients with an intermediate pretest probability of hepatic dysfunction, where it may help guide follow-up intensity or management decisions, and provides additional information when disease probability is very low or very high.^16^^,^^37^ Prior studies have similarly reported strong associations between HBP enhancement and liver function tests, highlighting the impact of scanner-dependent variability on spleen-referenced ratios and supporting the use of liver-only quantitative metrics and MRI-derived predictors of decompensation risk. These findings reinforce the expanding role of gadoxetic acid–enhanced MRI as both a morphologic and functional assessment modality.^16^^,^^17^^,^^30^^,^^37^^-^^39^

This study had several limitations. First, the retrospective, single-center design and exclusion of more than half of the initially screened patients introduce potential selection bias and limit the generalizability of the findings. Second, histopathological confirmation was lacking in most patients, preventing a direct correlation between LER and fibrosis stage or inflammatory activity. Third, all examinations were performed on a single 3.0-T scanner using a specific protocol; therefore, external validation across vendors and field strengths is required. Fourth, the sample size was insufficient to robustly assess etiology-specific differences; exploratory analyses by etiology (e.g., viral hepatitis vs. NAFLD vs. other causes) suggested similar trends but were underpowered and should be interpreted cautiously.

In conclusion, LER derived from gadoxetic acid–enhanced MRI is a reproducible, physiology-based imaging biomarker of hepatic function. It decreases with worsening Child–Pugh class and shows moderate inverse correlations with MELD and ALBI scores, providing clinically applicable cutoffs for detecting CLD and assessing disease severity. Given its modest sensitivity, ceiling effects in advanced disease, and single-center design, LER should not be used in isolation; rather, it may serve as a complementary tool to established laboratory scores to strengthen risk stratification and guide follow-up in CLD.

## Figures and Tables

**Table 1.** Demographic and Etiologic Characteristics of the Study Population

Variable	CLD (n = 78)	Controls (n = 109)	*P*
Age (years), median (IQR)	57.5 (47.0-62.8)	51 (42.0-59.0)	.17
Sex, n (%)			.08
Male	45 (57.7)	49 (45.0)	
Female	33 (42.3)	60 (55.0)	
Etiology of CLD, n (%)			
Hepatitis B virus infection	29 (37.2)	—	—
NAFLD	18 (23.1)	—	—
Hepatitis C virus infection	11 (14.1)	—	—
Alcoholic liver disease	7 (9.0)	—	—
Autoimmune/cholestatic liver disease (e.g., PBC, AIH, and PSC)	6 (7.7)	—	—
Cryptogenic or mixed etiology	7 (9.0)	—	—

AIH, autoimmune hepatitis; CLD, chronic liver disease; IQR, interquartile range; NAFLD, nonalcoholic fatty liver disease; PBC, primary biliary cholangitis; PSC, primary sclerosing cholangitis.

**Table 2.** Laboratory and Clinical Characteristics of Patients with CLD

Parameter	Median (IQR)
Bilirubin (mg/dL)	1.42 (0.86-2.41)
AST (U/L)	35 (23-55)
ALT (U/L)	31.5 (23-39.3)
GGT (U/L)	53 (26-108)
ALP (U/L)	98 (75-157)
Platelet count (×10^9^/L)	115 (76.5-145)
INR	1.25 (1.11-1.39)
Albumin (g/L)	40.1 (34.9-44.7)
MELD score	11 (9-13)
FIB-4 index	3.31 (1.87-5.71)

ALP, alkaline phosphatase; ALT, alanine aminotransferase; AST, aspartate aminotransferase; CLD, chronic liver disease; FIB-4, fibrosis-4 index; GGT, gamma-glutamyl transferase; INR, international normalized ratio; IQR, interquartile range; MELD, model for end-stage liver disease.

**Table 3.** LER According to Liver Function Categories

Group	n	LER, median (IQR)
Controls	109	1.29 (0.99-1.61)
CLD (all)	78	0.72 (0.54-1.05)
Child–Pugh A	51	0.86 (0.63-1.17)
Child–Pugh B	21	0.60 (0.38-0.79)
Child–Pugh C	6	0.42 (0.17-0.49)
ALBI grade 1	36	1.04 (0.74-1.23)
ALBI grade 2	36	0.58 (0.41-0.73)
ALBI grade 3	6	0.52 (0.41-0.62)
MELD <10	28	0.95 (0.70-1.16)
MELD 10-19	46	0.62 (0.45-0.90)
MELD ≥20	4	0.36 (0.17-0.51)

ALBI, albumin–bilirubin; CLD, chronic liver disease; IQR, interquartile range; LER, liver enhancement ratio; MELD, model for end-stage liver disease.

**Table 4.** Diagnostic Performance of the Liver Enhancement Ratio for Distinguishing Chronic Liver Disease Severity^a^

Comparison	AUC (95% CI)	Optimal LER cutoff	Sensitivity, % (95% CI)	Specificity, % (95% CI)
Healthy vs. chronic liver disease	0.83 (0.76-0.89)	1.01	57.7 (46.2-68.7)	90.8 (83.6-95.2)
ALBI grade ≥2 vs. grade 1	0.84 (0.77-0.89)	0.72	76.2 (63.8-86.0)	80.6 (68.4-89.0)
Child–Pugh B/C vs. A	0.78 (0.69-0.85)	0.62	70.4 (55.3-82.9)	78.4 (65.3-88.1)
MELD ≥10 vs. MELD <10	0.73 (0.65-0.82)	0.62	56.0 (41.3-69.8)	86.0 (75.3-93.0)

ALBI, albumin–bilirubin; AUC, area under the receiver operating characteristic curve; LER, liver enhancement ratio; MELD, model for end-stage liver disease.

^a^Data are presented as AUC with 95% CI. The 95% CIs were calculated using the DeLong method for AUC and Wilson score intervals for sensitivity and specificity.

**Table 5.** Correlation Between Liver Enhancement Ratio and Liver Function Scores

Score/Index	*ρ* (Spearman)^a^	*P*
MELD	−0.439	<.001
ALBI	−0.58	<.001
FIB-4	−0.421	<.01

ALBI, albumin–bilirubin; FIB-4, fibrosis-4 index; MELD, model for end-stage liver disease.

^a^*ρ* indicates Spearman’s rank correlation coefficient.
